# Embolization of Large Internal Iliac Artery Pseudoaneurysm through a Retrograde Trans-Superior Gluteal Arterial Access

**DOI:** 10.3390/tomography8050177

**Published:** 2022-08-24

**Authors:** Mohammad Ghasemi-Rad, Harshna V. Vadvala, Christie M. Lincoln, Zubin Irani

**Affiliations:** 1 Department of Radiology, Baylor College of Medicine, 1 Baylor Plaza, Houston, TX 77030, USA; 2 Department of Radiology and Radiological Science, Johns Hopkins Hospital, Johns Hopkins University, 601 N. Caroline Street, Baltimore, MD 21287, USA; 3 Department of Interventional Radiology, Massachusetts General Hospital, Harvard Medical School, 55 Fruit St., Boston, MA 02114, USA

**Keywords:** pseudoaneurysm, internal iliac, superior gluteal artery, retrograde, access

## Abstract

The presence of osteal stenosis/occlusion or osteal exclusion by prior interventions poses a challenge to selective catheterization of the internal iliac artery. We describe a case where a retrograde access through the superior gluteal artery (SGA) was used to successfully treat an internal iliac artery pseudoaneurysm (PSA) in a patient when an antegrade catheterization was not feasible due to internal iliac osteal exclusion by an endograft.

## 1. Introduction

The mechanism by which internal iliac artery (IIA) aneurysms develop is either acquired (trauma, surgery, biopsy, radiation) or from atherosclerosis. While the prevalence of non-traumatic IIA aneurysms in patients with aortoiliac atherosclerotic disease is 0.3%, there remains a dearth of data on the prevalence of traumatic IIA pseudoaneurysms in the literature. Most cases of IIA artery aneurysms occur in conjunction with aortic aneurysms [1]. The current recommendation is that an isolated iliac artery aneurysm that is larger than 3 cm should be treated. If the IIA aneurysm is identified concomitantly with an aortic aneurysm, then it can be treated at an even smaller size [2]. If not recognized or treated in a timely manner, the consequences can be devastating because the risk of IIA aneurysm rupture is 40–50% with a mortality of up to 80% [3,4]. All traumatic PSAs need to be treated as soon as possible due to the high risk of rupture and mortality associated with their rupture. 

We report on an alternative access through the left superior gluteal artery in a patient with an occluded left internal iliac artery. No institutional review board (IRB) approval was required for this case report.

## 2. Case Presentation

**Figure 1:**
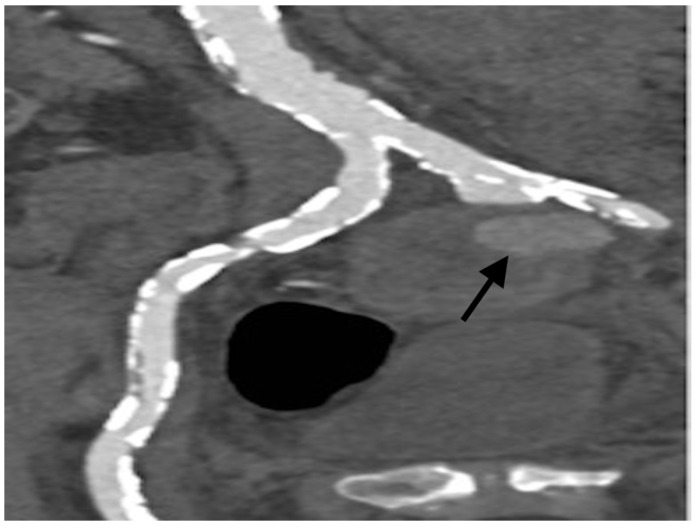
Coronal CTA with contrast demonstrating a PSA (black arrow) arising from the left internal iliac artery.

A 79-year-old male was found to have an incidental 7.3 cm large left internal iliac artery PSA during his ED presentation for trauma (fall from roof). The patient was stable on presentation. Surgical endovascular repair was carried out with balloon expandable endograft deployment into the left common iliac artery in an attempt to exclude and treat the PSA by vascular surgery; however, cannulation of the occluded left internal iliac artery was unsuccessful. A repeat contrast-enhanced CT scan showed the origin of the occlusion of the left internal iliac artery, immediately proximal to the PSA (Figure 1), and a prominent superior gluteal artery as a potential feeder. Given persistent filling of the PSA via retrograde collaterals, he was referred to interventional radiology for a PSA embolization attempt.

**Figure 2:**
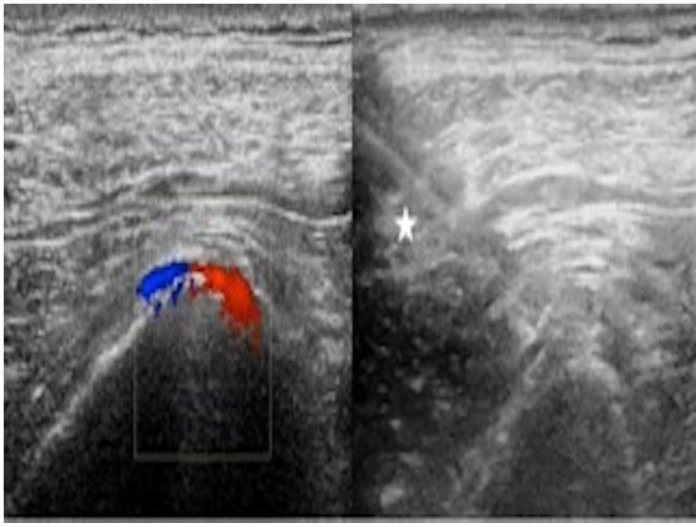
Color Doppler and gray-scale ultrasound of the gluteal region demonstrating patent superior gluteal artery that was accessed under ultrasound guidance (*).

Ultrasound of the left gluteal region was carried out, and an access site for puncture of the superior gluteal artery (SGA) was identified. Under ultrasound guidance, the left superior gluteal artery (SGA) was accessed with a 21-gauge, 5 cm needle and upsized to a 4 French micropuncture system using the Seldinger technique (Figure 2, star—needle access).

**Figure 3:**
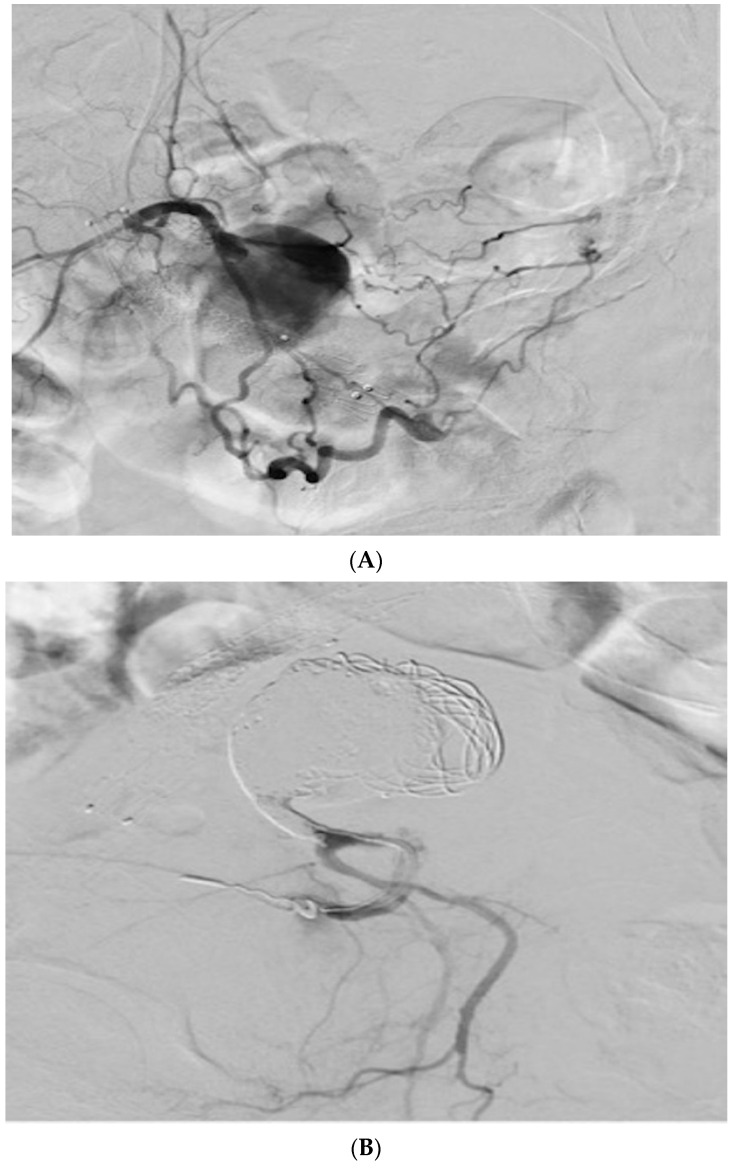
(**A**). Initial angiogram demonstrated a large PSA arising from the left internal iliac artery. (**B**). Post-coil embolization angiogram demonstrated non-opacification of the aneurysm.

Subsequent digital subtraction angiography through the 4 F sheath demonstrated filling of a large internal iliac PSA (Figure 3A). 

A 0.035 in glidewire was advanced through the 4 F micropuncture sheath, and the micropuncture system was exchanged for a 5 F Kumpe catheter. Hand injection of contrast confirmed positioning of the catheter within the PSA. The PSA was coiled via a 5 French Kumpe catheter with variety of 0.035 coils. Post-embolization angiogram demonstrated complete exclusion of PSA with no flow (Figure 3B).

**Figure 4:**
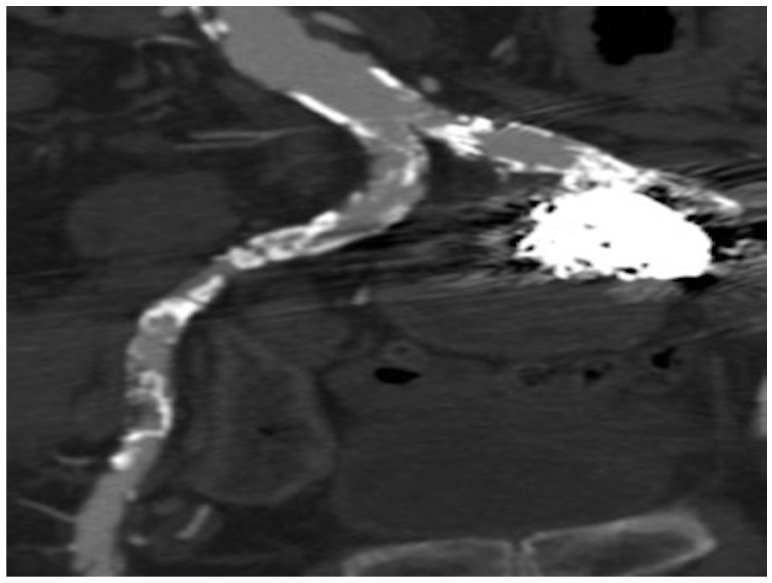
Post-embolization follow-up coronal CTA with contrast demonstrated complete closure of the pseudoaneurysm.

At completion of the procedure, a Gelfoam–thrombin mixture was injected while pulling out the catheter to the point of reflux proximal to the puncture point into the SGA to reinforce hemostasis. A 5-week follow-up CTA showed a smaller PSA without contrast filling (Figure 4). 

A 1-year follow-up CT showed complete resolution of the PSA with no contrast filling. Written informed consent was obtained from the patient’s family member.

## 3. Discussion

There are no reports of the prevalence of traumatic PSAs of the IIA in the literature. IIA aneurysms/PSAs are usually asymptomatic but can present with non-specific symptoms such as pelvic heaviness and can even compress adjacent structures such as nerves, form fistulae or rupture as an initial presentation. 

Treatment choices for IIA aneurysms/PSAs are open surgery (resection of the aneurysm, ligation of the aneurysm proximally, or proximal ligation with endoaneurysmorrhaphy), an endovascular approach or a hybrid of both the endovascular and surgical approaches [2]. Surgical options are usually challenging especially in cases of distal IIA aneurysms, previous pelvic surgery or endovascular aortic grafts due to scaring as well as a change in anatomy [5]. Open procedures are usually morbid, and many patients are not good candidates for a substantial procedure [6]. 

With the advent of interventional techniques, the endovascular approach has been the mainstay of treatment, and the hybrid approach is employed for more complex cases. The endovascular approach, with the exclusion of the aneurysm sac from antegrade and retrograde perfusion, is proved to be a feasible treatment option. However, the percutaneous approach becomes limited when the internal iliac artery access is no longer possible either due to prior surgical ligation (surgical aortoiliac reconstruction), jailing due to stenting (pre-existing endovascular aneurysm repair) or severe stenosis in the elderly population with severe atherosclerotic disease [7]. The best endovascular treatment should embolize all proximal feeding vessels as well as all distal runoff branches [1]. Otherwise, the aneurysm sac will again fill and increases the risk of rupture. In such instances, there are limited endovascular options to embolize the distal runoff branches. If the aneurysm/PSA and distal feeders are incompletely embolized, there is an increased risk of aneurysm/PSA re-expansion [1]. The other potential treatment method is direct ultrasound or CT-guided puncture of the PSA with either thrombin or coil embolization of the aneurysm/PSA [8]. 

In the index case, the previous intervention rendered the transfemoral approach unachievable. Instead, we successfully embolized the IIA PSA through left SGA access. Gluteal arteries are usually the culprit vessels in the retrograde filling of IIA aneurysms as well as type II endoleaks after endovascular aortic repair [9]. A few similar published cases in the English-language literature include Chi et al., who reported endovascular treatment of a right IIA aneurysm in a jailed right IIA ostium using SMART-ultrasound-guided needle access to directly puncture the right SGA [10]. Herskowitz et al. described a patient with an isolated 3 cm left IIA aneurysm that doubled in size on the follow-up scan after 8 months. This group successfully accessed the left SGA with US and embolized the aneurysm sac as well as the feeders [1]. Thomas et al. also used SGA access under fluoroscopic guidance to embolize an endoleak after EVAR [7]. 

Parlani et al. recounted a patient with a prior bilateral common iliac artery stent graft and bilateral IIA aneurysm. They used contrast CT to access the bilateral SGA followed by coil embolization of the aneurysms [11]. Another report by Keagan Werner-Gibbing demonstrated the embolization of a type II endoleak with multiple collateral supplies with the SGA approach [12]. Magishi et al. also accessed the SGA for excluded IIA aneurysm repair after AAA; however, this was carried out through a surgical exploration of buttock and Seldinger access of the SGA followed by coil with fluoroscopic guidance utilizing both anatomic landmarks, and the road map after bilateral femoral access was inaccessible in a patient with previous aortoiliac bypass [6]. Rynio et al. developed a 3D model that can be employed to learn and be trained on gluteal artery access [9]. Lastly, Gemmete et al. shared an unusual transosseous direct puncture of an IIA aneurysm [13].

While the benefit of endovascular treatments is evident, this modality cannot remove the mass effect induced by giant aneurysms [2]. In addition, there are potential risks that include pelvic neurovascular and hollow organ injury, bleeding from access into the pelvis, pseudoaneurysm at the access site and buttock claudication [6].

All described cases of SGA were due to jailing of access through the IIA and inadequate embolization of distal aneurysm feeders [1,10]. In our index case, the utilization of percutaneous retrograde access under ultrasound guidance helped to prevent open surgery. 

## 4. Conclusions

Our case demonstrates that SGA access is a feasible and safe alternative technique when conventional endovascular approaches are unattainable.

## Data Availability

Not applicable.
